# Facile Formation of Metallic Surface with Microroughness via Spray-Coating of Copper Nanoparticles for Enhanced Liquid Metal Wetting

**DOI:** 10.3390/ma17215299

**Published:** 2024-10-31

**Authors:** Ji-Hye Kim, Ju-Hee So, Hyung-Jun Koo

**Affiliations:** 1 Department of Chemical & Biomolecular Engineering, Seoul National University of Science and Technology, 232 Gongneung-ro, Nowon-gu, Seoul 01811, Republic of Korea; gh5289@naver.com; 2 Material & Component Convergence R&D Department, Korea Institute of Industrial Technology, Ansan-si 15588, Republic of Korea

**Keywords:** liquid metal, wetting, imbibition, spray coating, copper nanoparticles

## Abstract

This paper presents a simple, fast, and cost-effective method for creating metallic microstructured surfaces by spray-coating a dispersion of copper nanoparticles (CuNPs) onto polymethyl methacrylate (PMMA) substrates, enabling the imbibition-induced wetting of liquid metal. The formation of these microstructured patterns is crucial for the spontaneous wetting of gallium-based liquid metals. Traditional techniques for producing such microstructures often involve complex and costly lithography and vacuum deposition methods. In contrast, this study demonstrates that liquid metal wetting can occur with metal microstructures formed through a straightforward spray-coating process. To immobilize the CuNPs on the polymer substrate, an organic solvent that dissolves the polymer surface was employed as the dispersion medium. The effects of various spray-coating parameters, including distance and time, on the uniformity and immobilization of CuNP films were systematically investigated. Under optimal conditions (120 s of spray time and 10 cm spray distance), CuNPs dispersed in dichloromethane (DCM) yielded uniform and stable microstructured surfaces. The spontaneous wetting of gallium-based liquid metal was observed on the fabricated CuNP film. Additionally, liquid metal selectively wet the CuNP patterns formed by stencil techniques, establishing electrical connections between electrodes. These findings underscore the potential of spray-coating for fabricating metallic surfaces to drive the formation of liquid metal patterns in flexible electronics applications.

## 1. Introduction

Gallium-based liquid metal alloys (GaLMs) have garnered significant interest due to their attractive properties, including a low melting point, high electrical conductivity, low viscosity, fluidity, low toxicity, and high deformability [1,2]. These characteristics make GaLMs exceptionally suitable for applications requiring stretchable and deformable electronic pathways, such as flexible electronics [3,4,5,6,7], soft robotics [8,9,10], biomedical devices [11], and energy harvesting systems [12,13,14]. The most commonly used GaLMs are eutectic alloys of gallium–indium (EGaIn) and gallium–indium–tin (Galinstan).

GaLMs exhibit exceptionally high surface tension. Although the formation of an oxide film on the surface can effectively reduce the surface tension, it remains challenging to reproducibly print or form thin films of these liquid metals. One of the most effective methods for reliably printing GaLMs onto a substrate is by leveraging the phenomenon of reactive wetting. Liquid metals readily react with many solid metals to form alloys, thereby creating a stable interface [15,16,17]. By exploiting the favorable wetting properties of GaLMs on metallic surfaces, it is possible to achieve the reproducible coating or patterning of these materials [18,19,20]. Moreover, introducing surface roughness to the metallic substrate can further enhance the wetting characteristics of GaLMs. Our research has demonstrated that on metallic substrates with well-defined microstructures, the wetting properties of GaLMs are significantly improved by a capillary-driven phenomenon known as imbibition [21]. This imbibition-induced wetting occurs rapidly and spontaneously, enabling GaLMs to form flat films through complete wetting. These findings are poised to enhance the processability of liquid metals, expanding their applicability across a broader range of applications. However, this method necessitates the use of lithography and vacuum deposition techniques to fabricate microstructured metallic substrates, which introduces several challenges. These processes are not only time-consuming and costly, but they also involve intricate procedures that require specialized equipment and expertise. Furthermore, the reliance on these techniques may impose limitations on the types of materials that can be used as substrates, potentially restricting the versatility and scalability of the approach. Such constraints could hinder the broader adoption and application of the imbibition-induced wetting in various fields where diverse material compatibility is essential.

**Figure 1:**
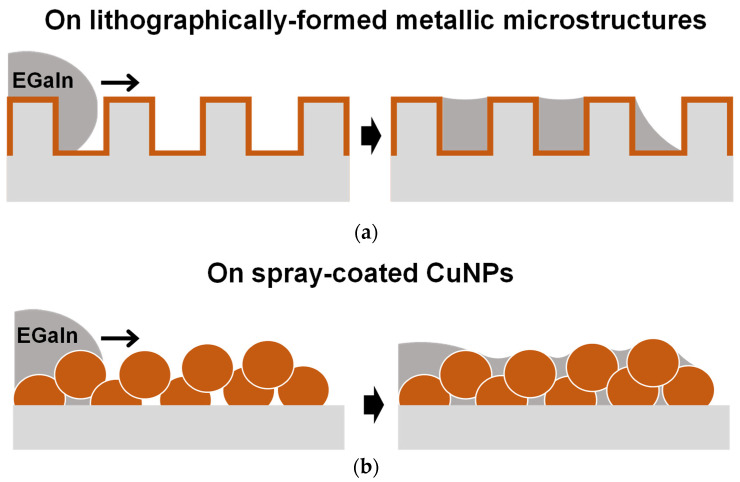
Schematics depicting the imbibition-induced wetting behaviors of liquid metal on a microstructured metallic surface formed by different processes: (**a**) fabricated by the lithography process and vacuum metal deposition and (**b**) formed by the one-step spray coating of CuNPs.

Herein, as a simpler and more cost-effective approach to form the microstructured metallic surfaces required for the imbibition-induced wetting of liquid metals, dispersions of Cu nanoparticles (CuNPs) are spray-coated onto polymer substrates (Figure 1). By spraying CuNPs, the deposited CuNP film can have an inherent microstructure. The spraying process is performed at room temperature and atmospheric pressure and is fast and economical. For the enhanced wetting of liquid metal induced by imbibition, the spray-coated CuNPs should form a uniform film with well-connected particles and be firmly and reliably anchored to the substrate. To immobilize the spray-coated CuNPs on the polymer substrate, organic solvents capable of dissolving the polymer are utilized as the solvent of the CuNP spray dispersion [22,23,24]. For the formation of CuNP films with complete surface coverage and strong adhesion, we systematically investigate the influence of various spray conditions, such as the type of dispersion solvent and spray distance/time, on the uniformity and stability of the resulting CuNP films. On the CuNP films prepared with optimal spray coating conditions, liquid metals show enhanced wetting and spreading induced by the imbibition principle. Finally, we demonstrate the selective wetting of liquid metal on patterned CuNP films fabricated by the stencil method.

## 2. Materials and Methods

### 2.1. Materials

CuNPs (diameter: ~500 nm) were purchased from SkySpring Nanomaterials, Houston, TX, USA. Acetone, isopropyl alcohol (IPA), dichloromethane (DCM), dimethyl sulfoxide (DMSO), and hydrochloric acid (HCl, 37 wt.%) were purchased from Samchun Chemicals, Seoul, Republic of Korea. Chlorobenzene (CB), *N*,*N*-dimethylformamide (DMF), and EGaIn (gallium 75.5%, indium 24.5%, >99.99%) were purchased from Sigma-Aldrich, St. Louis, MO, USA. Poly(methyl methacrylate) (PMMA) substrates were purchased from Acrylmall, Incheon, Republic of Korea.

### 2.2. Spray Coating of Copper Nanoparticles

The CuNPs were dispersed in various solvents, i.e., acetone, IPA, DCM, CB, DMSO, and DMF (20 mg/mL), and tip-sonicated (GM2070, Bandelin, Berlin, Germany) for 5 min to improve the dispersion homogeneity of the CuNPs before spray coating. The dispersions were sprayed by the spray gun (GP-70, nozzle size: 0.7 mm, SPARMAX, Taipei, Taiwan) on PMMA substrates (25 mm × 25 mm, Acrylmall, Incheon, Republic of Korea), as shown in Figure S1, at various distances (5 cm, 10 cm, and 20 cm) from the substrate. The spray angle was 40° and the pressure of the air compressor was 1 bar. The spray gun was fixed to a stand to keep the spray angle and distance constant, which is important for stable particle deposition. CuNP films were patterned by spraying CuNP dispersion through stencil masks.

### 2.3. Wetting of EGaIn Liquid Metal

The substrates coated with the CuNPs were placed in a 5 cm × 5 cm × 5 cm glass chamber (Hellma USA Inc., New York, NY, USA), and 4-5 μL of EGaIn droplets were placed on the substrate. To remove the gallium oxide layer of EGaIn, a 20 μL drop of HCl solution was placed next to the substrate. As the HCl solution evaporates, the oxide on the liquid metal was removed within 10 s and the wetting of the EGaIn began.

### 2.4. Characterization

The surface morphology and structure of the CuNPs coated PMMA substrates (CuNPs/PMMA) were characterized by an optical microscope (BX51M, OLYMPUS, Tokyo, Japan) and a scanning electron microscope (SEM, JSM-6700F, JEOL Ltd., Tokyo, Japan) with a secondary electron (SE) detector (JEOL Ltd., Tokyo, Japan) at 10 kV and a working distance of 8 mm. For the cross-sectional SEM images, the CuNPs/PMMA was cut using a PMMA cutter (RunXin Industry, Nanjing, China) and then sputter-coated with platinum. For the stability tests, the central area of the spray-coated CuNPs/PMMA film was gently swabbed with a cotton swab to assess the adhesion between the PMMA substrate and CuNPs. The surface coverage values were obtained from optical microscope images using ImageJ software (Version 1.54g, National Institutes of Health, Bethesda, MD, USA). The images were first converted to 8-bit format and a threshold was applied without a dark background. The area fraction of the thresholded images was then measured, and the average area fraction was calculated for multiple regions within each sample. In the experiment to investigate the change in the electrical resistance of the CuNPs/PMMA as EGaIn wetting proceeds, the CuNPs film (width = 3 mm, length = 1 cm) was prepared by spray-coating with 120 s of spray time and 10 cm of spray distance through a stencil mask. The electrical current was measured over time using a Keithley 2450 source meter (Keithley, Solon, OH, USA).

## 3. Results

**Figure 2:**
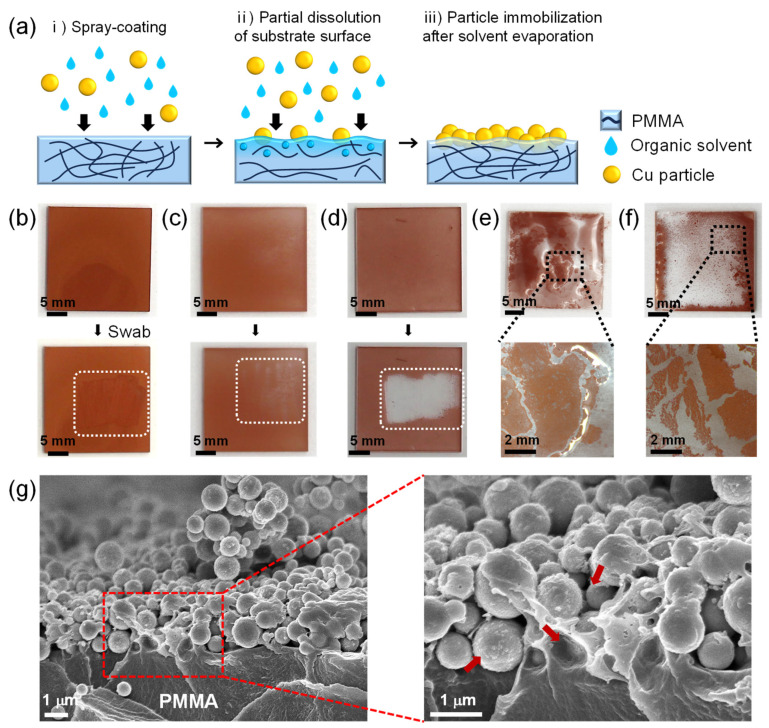
(**a**) Schematic of the film formation of the CuNPs immobilized on the PMMA via spray-coating method. (**b**–**d**) Film stability of the CuNPs on the PMMA substrate with different dispersion solvents: (**b**) DCM, (**c**) CB, and (**d**) IPA. The swabbed areas are marked as white dotted rectangles. When the CuNPs are peeled off, as in (**c**), the white background is revealed through the transparent PMMA layer. (**e**,**f**) Optical images of CuNPs/PMMA substrate after spraying CuNP dispersions, using (**e**) DMSO and (**f**) DMF as dispersion solvents. (**g**) Cross-sectional SEM images of the CuNPs/PMMA substrate using DCM as the dispersion solvent at low and high magnification. The red arrows indicate the areas of PMMA that are dissolved by DCM and anchoring the CuNPs. For all samples, 20 mg/mL of the CuNP dispersions were sprayed for 60 s at the spray distance of 10 cm.

In order for the deposited CuNPs to maintain their microstructure during the wetting of the liquid metal, the CuNPs should be stably anchored and immobilized to the substrate. Figure 2a illustrates the spray-coating method of CuNPs immobilized onto a PMMA substrate, where a micro-textured metallic surface is formed for the imbibition-induced wetting of GaLMs. PMMA was chosen as a model material for the substrate because it is inexpensive and lightweight with high transparency and excellent processability. To immobilize the spray-coated CuNPs on the substrate, an organic solvent highly compatible with the surface of the polymer substrate is employed as a medium for the CuNP dispersion. During the spray-coating process, the solvent wets the polymer surface, partially dissolving or swelling the polymer to form a gel-like layer on the top. Concurrently, the spray-coated CuNPs partially penetrate into the polymer surface. As the solvent evaporates, the gel-like layer solidifies and the CuNPs are immobilized on the polymer substrate.

For the efficient immobilization of CuNPs on the substrate, the solvents of the CuNP dispersion should be carefully selected to be ones that evaporate well while adequately dissolving or swelling the substrate. To investigate the effect of dispersion solvents on the particle immobilization on the PMMA substrate, five different solvents—DCM, CB, IPA, DMSO and DMF—were used for CuNP dispersions, which were sprayed onto the PMMA substrates (Figure 2b–f). After the spray-coating of the dispersions followed by drying at room temperature for 5 min, the coated CuNP films were swabbed with a cotton swab to determine whether the CuNPs were firmly immobilized. When DCM or CB, which can dissolve PMMA, are used as the solvent, the CuNPs are not easily removed by swabbing with a cotton swab, indicating that the metal particles are effectively immobilized on the PMMA substrate (Figure 2b,c and Video S1). Figure 2g shows the cross-sectional SEM images at the interface of the PMMA substrate and the spray-coated CuNPs when DCM is used as the dispersion solvent. It is observed that the CuNPs are well embedded and immobilized within the dissolved surface of the PMMA substrate. In contrast, in the case of IPA, which is commonly used as a solvent for nanoparticle dispersions [25,26,27,28], the particles were easily peeled off from the substrate (Figure 2d and Video S2). This is because PMMA is almost insoluble in IPA, so the copper particles simply sit on the substrate without immobilization. For the uniform coating of CuNPs, it is also necessary to select a solvent with appropriate volatility. DMSO and DMF are also solvents that can dissolve PMMA, but due to their low vapor pressure (Table S1), they do not evaporate sufficiently during the spray-coating process, causing the dispersion to pool on the substrate. As a result, the particles floated in the solutions and agglomerated, forming a non-uniform CuNP film, as shown in Figure 2e,f.

**Figure 3:**
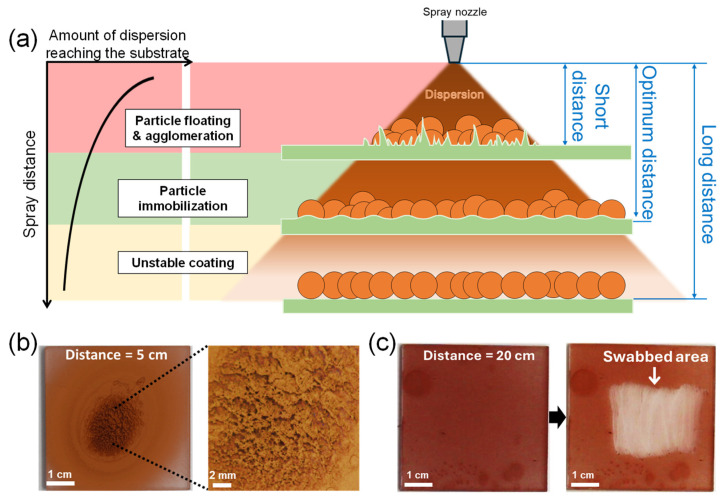
(**a**) Schematic illustrating the relationship between spray distance, the amount of dispersion reaching the substrate, and the resulting film state of spray-coated nanoparticles. (**b**,**c**) Optical images of the surface of CuNPs/PMMA substrates sprayed at different spray distances: (**b**) 5 cm (short distance) and (**c**) 20 cm (long distance) from the substrate. The dispersion solvent is DCM. The high-resolution image on the right in (**b**) shows agglomerated CuNPs after drying. (**c**) Coating stability of the CuNPs/PMMA substrate sprayed at a distance of 20 cm from the substrate. The image on the right in (**c**) shows the particles peeling off when wiped with a cotton swab, indicating that the CuNPs are not properly immobilized on the substrate.

In addition to selecting the appropriate dispersion solvent, the “spray distance”, the distance from the spray nozzle to the substrate, is crucial for particle immobilization [29,30]. Figure 3a depicts the expected relationship between the spray distance, the amount of dispersion reaching the substrate, and the resulting film state of spray-coated nanoparticles. If the spray distance is too short, the dispersion sprays in a small, focused area and pools on the substrate surface. As a result, the sprayed CuNPs float and agglomerate in the excess solvent, preventing them from being uniformly deposited on the substrate. If the spray distance is too long, the dispersion could cover the substrate evenly, but the solvent may evaporate midway before reaching the substrate and the particles may not be immobilized.

Figure 3b,c compare the film morphology and stability of the CuNPs coated on PMMA substrates at spray distances of 5 cm and 20 cm. When the spray distance is 5 cm (Figure 3b), the spray-coated CuNPs are concentrated in a small area in the center. Although the CuNPs are immobilized after drying, the coated surface is uneven and rough due to particle agglomeration. Conversely, when the spray distance is relatively long, i.e., 20 cm from the substrate (Figure 3c), the spray-coated CuNPs are not well immobilized on the PMMA substrate and are easily swabbed off. This result implies that under the conditions of this experiment (dispersion concentration, solvent, spray angle, spray pressure, etc.), the spray distance should be longer than 5 cm and shorter than 20 cm for the uniform coating and immobilization of CuNPs, as shown in Figure 2b,c, where the spray distance is 10 cm. The appropriate spray distance should depend on the spray conditions. Therefore, to obtain a uniform and stable particle film, it is important to set the optimal spray distance for each spray condition, based on the principle described in Figure 3a.

**Figure 4:**
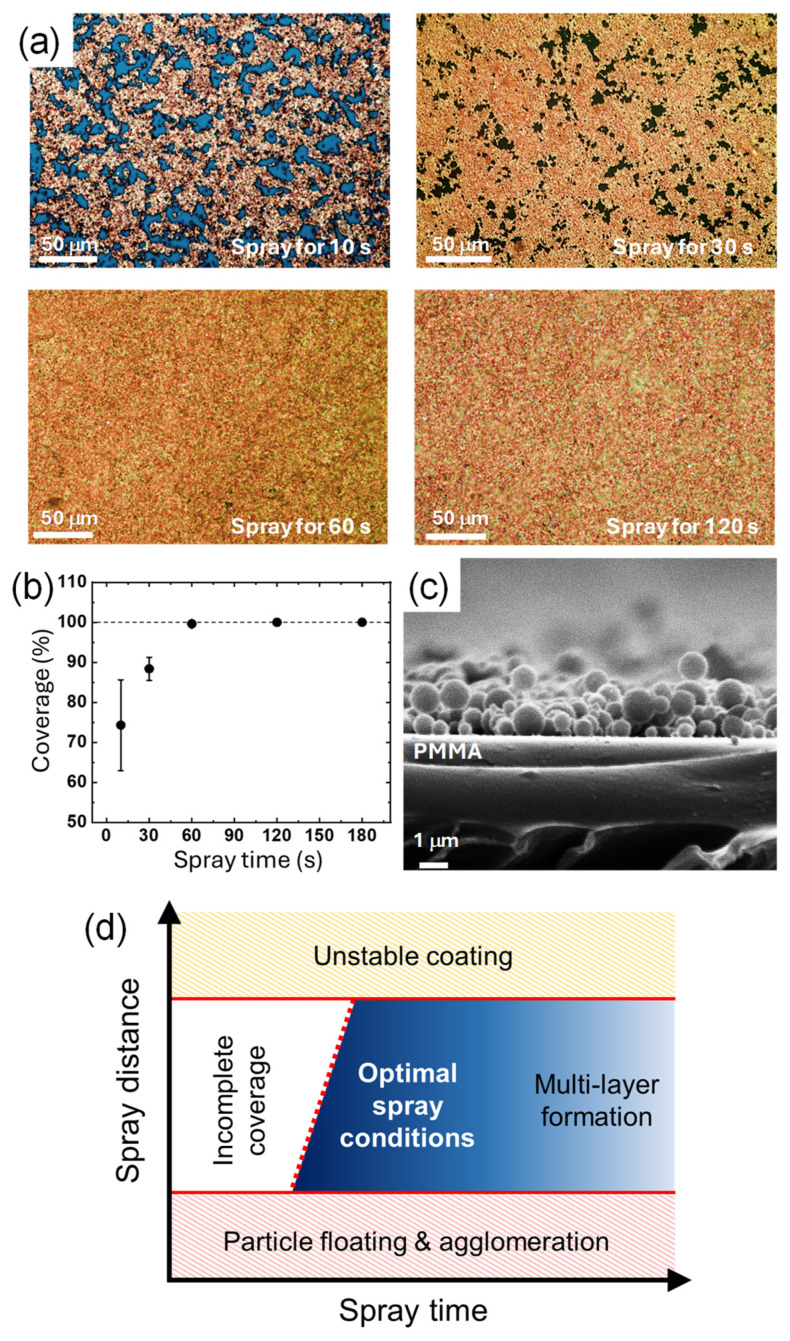
(**a**) Optical images of the surface of the CuNPs/PMMA substrates with different spray times. (**b**) Surface coverage of the CuNPs/PMMA depending on the spray time. (**c**) Cross-sectional SEM image of the CuNPs/PMMA with the spray times of 10 s. The spray distance was fixed at 10 cm, and DCM was used as the solvent of the CuNP dispersion. (**d**) A schematic diagram of the states of the CuNP film as a function of the two spray parameters of time and distance.

Besides the spray distance, the spray time also affects the amount of CuNPs deposited and the amount of solvent, which in turn affects the resulting film state of CuNPs. To compare the amount of the particles deposited, the surface morphology of the CuNPs/PMMA substrates is compared when the CuNP dispersion is sprayed for 10 s, 30 s, 60 s, and 120 s (Figure 4a). When the spray time is less than 30 s, the PMMA substrate is not completely covered with the CuNPs and scattered voids are observed. As the spray time increases to 60 and 120 s, the surface coverage increases, and no noticeable voids are observed. For quantitative analysis, the coverage values calculated using ImageJ software were plotted as a function of the spray time (Figure 4b). In 10 s, about 75% of the surface area of the substrate is covered with CuNPs, and almost 90% of the surface is covered in 30 s. The plot shows that the coverage increases with the spray time and saturates to 100% at 60 s, indicating that the PMMA substrate is completely covered with the CuNPs without any voids. At spray times longer than 60 s, the coverage remains almost constant, although multilayers of CuNPs may form on the substrate.

A sufficient spray time is also required to supply a sufficient amount of solvent to the substrate. In order for the solvent to partially dissolve the PMMA substrate and immobilize the CuNPs as shown in Figure 2g, a sufficient amount of solvent should be supplied. For example, for the PMMA substrate coated for only 30 s (Figure 4c), the CuNPs are not embedded in the substrate and simply sit on the surface of the substrate. In this case, it is difficult for the particles to be firmly anchored to the substrate. Similar to the spray distance condition, the appropriate spray time can vary depending on the spray conditions. Therefore, the spray time should also be adjusted to ensure that a sufficient amount of solvent and CuNPs are applied on the substrate to effectively coat and immobilize the particles. The states of the CuNP film as a function of the two spray parameters considered in this study are summarized in the schematic diagram shown in Figure 4d. To achieve a uniform monolayer of CuNPs stably immobilized on the surface, optimal spray time and spray distance should be applied. The red dotted line in the diagram indicates that the spray time required to obtain a CuNP film with complete coverage increases with increasing spray distance, reflecting the fact that a greater distance reduces the amount of CuNPs deposited per unit area. Thus, spray distance and time are interdependent factors that should be considered when determining optimal spray conditions. In addition to spray distance and time, various other spray conditions, such as spray pressure, physicochemical properties of the substrate and solvent, ambient temperature, etc., may interact with each other in the spray process. For example, if the ambient temperature is higher, the solvent may be more prone to volatilization, which may require a shorter spray distance. A systematic study of the mutual influence of these various spray process parameters is currently under investigation.

**Figure 5:**
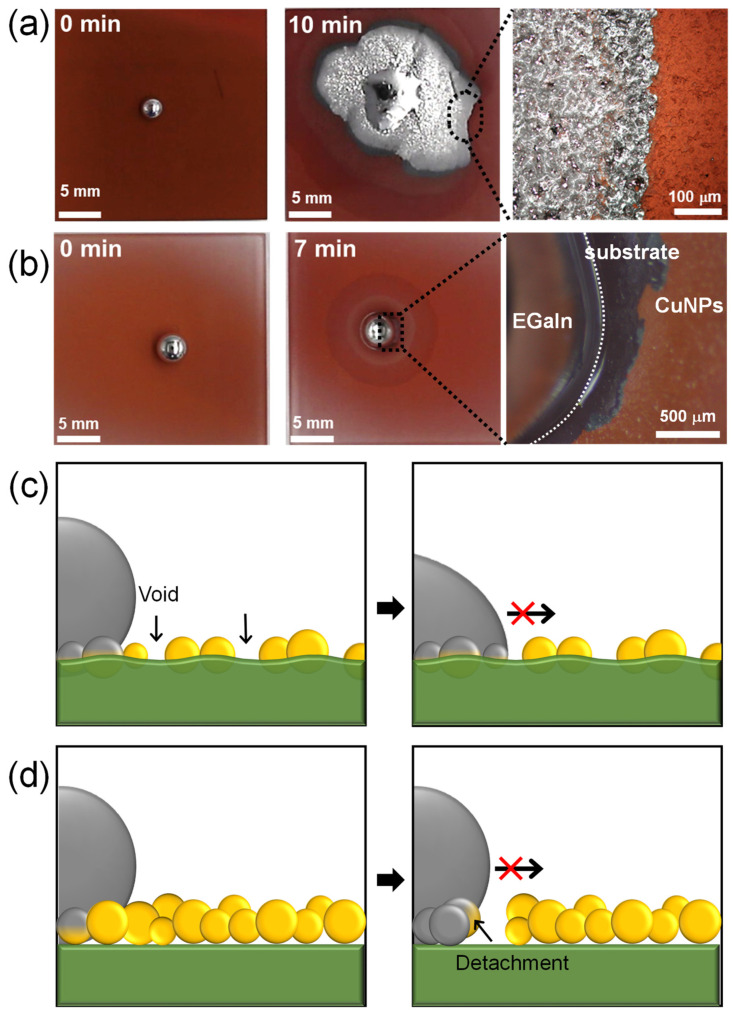
Wetting behavior of EGaIn liquid metal on CuNPs/PMMA substrate with spray times of (**a**) 120 s and (**b**) 30 s. (**c**,**d**) Schematic illustrating the incomplete imbibition-induced wetting of liquid metal on CuNP/PMMA substrates formed at low spray time: (**c**) CuNPs are immobilized on the substrate, but the coverage is insufficient, preventing the imbibition of EGaIn. (**d**) CuNPs are not immobilized on the PMMA substrate, resulting in the detachment of the CuNPs.

Figure 5a,b show the wetting behavior of liquid metal on spray-coated CuNP films. Based on the results obtained from varying the spray distance and time, the CuNPs/PMMA substrates were prepared using the optimal spray distance of 10 cm and spray times of 30 or 120 s. According to our previous research, the imbibition of EGaIn liquid metal occurs when an EGaIn liquid metal droplet placed on a metallic rough surface is exposed to HCl vapor. This exposure removes the gallium oxide layer and promotes reactive wetting between the EGaIn and metal surface. On the CuNPs/PMMA with the spray time of 120 s, the enhanced wetting of liquid metal is shown where capillary-driven imbibition behavior is observed at the edge (Figure 5a and Video S3). However, on the CuNPs/PMMA with the spray time of 30 s, liquid metal beads up due to the high surface tension and no wetting behavior is observed (Figure 5b and Video S4). There are two main reasons why the imbibition-induced wetting behavior of liquid metals is not observed at low spray times: the low coverage of CuNPs and a weak adhesion between the CuNPs and the substrate (Figure 5c,d). Previous studies have reported that the stable imbibition of liquid metal on a post-patterned substrate requires that (1) the posts are sufficiently tall; and (2) the gap between the posts is sufficiently narrow [21,31]. This means that the gap between the metallic parts into which the liquid metal penetrates needs to be small enough. Since the CuNPs are not coated quite compactly or even voids form with the short spray time, as shown in Figure 4a, the wetting of the liquid metal cannot proceed. In addition, when the adhesion between the metallic particles and the substrate is weak, the particles may detach from the substrate during reactive wetting with the liquid metal [18]. In fact, some CuNPs were observed to detach from the substrate under the liquid metal droplets (Figure 5b). When the metal particles detach from the substrate, voids are formed, and capillary-driven imbibition wetting cannot occur.

**Figure 6:**
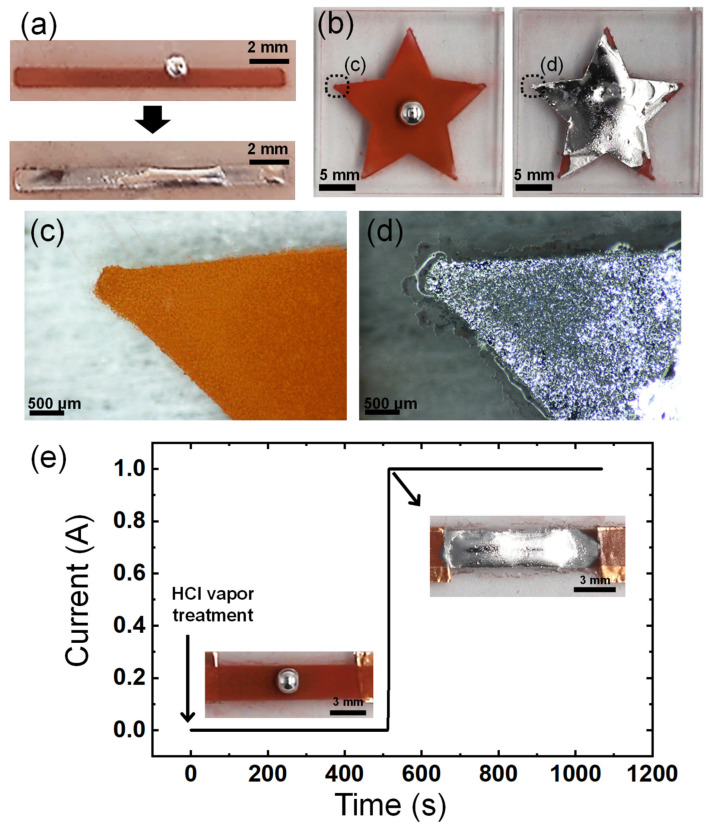
Patterning of the liquid metal via selective wetting. Imbibition-induced wetting of the liquid metal (**a**) on the line pattern with a width of 1 mm and (**b**) on a star-shaped pattern. (**c**,**d**) Magnified images of the edge of the star-shaped pattern in (**b**): (**c**) before and (**d**) after wetting of liquid metal. (**e**) Change in the current of the spray-coated CuNP film before and after EGaIn wetting.

As the imbibition-wetting of liquid metal occurs upon contact and reaction with CuNPs, the patterned CuNP films can be used to enable the selective wetting of liquid metals. The patterns of the CuNP films can be easily formed by spraying the CuNP dispersion through a stencil mask. Figure 6a,b show the selective imbibition occurring along the pattern of spray-coated CuNPs on a PMMA substrate. The selective wetting of EGaIn is observed in a narrow line pattern, as small as 1 mm wide. The imbibition of the liquid metal proceeds even at the sharp edges of the star, where the smallest dimensions are less than 500 μm (Figure 6c,d). The resolution of the pattern at the smallest size where the imbibition of the liquid metal can occur may depend on the size of the CuNPs or the precision of the spray-coating. The factors involved in the resolution of imbibition need to be studied in depth in the future. However, it is clear that the spray-coating of CuNPs offers a significantly simpler and faster method for patterning microstructured metallic surfaces compared to lithography and vacuum metal deposition processes, and as a result, the pattern of the liquid metal can be formed easily and quickly.

To demonstrate the potential application of the imbibition of the liquid metal to electronic devices, it was observed how the electrical conductivity of the line pattern of the CuNPs/PMMA changed over time as the imbibition of the liquid metal progressed. The plot in Figure 6e shows a very low electrical conductivity in the line pattern until the liquid metal wets entirely and connects the two ends of the pattern. There is a significant increase in electrical current by 12 orders of magnitude about 500 s after exposure to HCl vapor for imbibition (Figure S2). This sharp increase in current implies that the continuous electrical pathway in the liquid metal has a much lower resistance than the layer of CuNPs. The layer of CuNPs exhibits low electrical conductivity, possibly due to the presence of insulating layers such as an oxide shell or organic ligands on the particle surface, which inhibits effective electrical flow. To address this problem, an additional high-temperature sintering process, generally in the range of 250 °C to 350 °C, is often necessary to enhance conductivity [32,33,34]. However, the high-temperature process requires a lot of energy and is difficult to apply to substrates that are susceptible to high temperatures, such as polymers. The imbibition of the liquid metal can significantly increase the conductivity of the patterned CuNPs at room temperature. This demonstrates the potential and advantages of using selectively wet EGaIn in applications such as flexible electronic circuits and soft robotics composed of polymers, gels, elastomers, etc.

## 4. Conclusions

This paper presents a simple, fast, and cost-effective method for forming metallic microstructured surfaces by spray-coating CuNP dispersion on PMMA substrates for the imbibition-induced wetting of liquid metal. The formation of metallic microstructured patterns is essential for the spontaneous wetting of liquid metals onto copper solid metal substrates. In the previous studies, the metallic microstructures were designed to be regularly arranged and fabricated using complex lithographic processes. However, in this study, it is experimentally demonstrated that liquid metal wetting can occur in the irregular metal microstructures formed using a simple spray-coating process. Organic solvents such as DCM and CB, which can dissolve the PMMA substrates, were used as CuNP dispersion solvents to effectively immobilize and anchor the spray-coated CuNPs to the substrate. Under the optimal conditions of 120 s spray time and 10 cm spray distance, complete surface coverage and robust CuNPs adhesion to the substrate were achieved, as confirmed by optical microscopy, swab tests, and SEM analysis. Liquid metals exhibit spontaneous and selective wetting behavior on these CuNP patterns formed via the spray-coating method, facilitating the easy implementation of liquid metal patterning.

An important point is that various spray-coating parameters can be adjusted according to the chemical properties of the substrate and dispersion to form metallic microstructured surfaces. This paper is limited to analyzing an exemplary case of forming a metallic microstructured surface on a PMMA substrate using a spray-coating method. However, the discussed method can be applied not only to PMMA, but also to soluble organic substrates. Depending on the solubility of the substrate, a suitable dispersion solvent can be selected and the spray parameters can be adjusted. In addition to copper, the spray coating method can also be applied to dispersions of other metals that wet the liquid metals. The spray coating parameters would need to be controlled depending on the type and size of the metal particles and their wettability with the liquid metal. Furthermore, it is worthwhile to study the long-term durability of the liquid metal patterns on the fabricated metal microstructured surfaces under various conditions such as humidity, temperature, and stress for future research. In conclusion, this study presents a versatile method for fabricating metallic microstructured surfaces for the wetting of liquid metals and provides a basic guide for a wide range of applications including advanced flexible and stretchable devices, opening up new possibilities for future advancements in the field.

## Data Availability

The data in this study are available on request from the corresponding authors.

## Supplementary Materials

The following supporting information can be downloaded at https://www.mdpi.com/article/10.3390/ma17215299/s1: Figure S1: Schematic of the setup for CuNPs spray-coating; Figure S2: Log plot of change in the current of the spray-coated CuNP film before and after EGaIn wetting; Table S1: Vapor pressure, surface tension, and relative energy difference (RED) of organic solvent used for dispersion; Video S1: Film stability of CuNPs on the PMMA substrate using DCM and CB as dispersion solvent; Video S2: Film stability of CuNPs on the PMMA substrate using IPA as dispersion solvent; Video S3: Wetting behavior of EGaIn liquid metal on CuNPs/PMMA substrate with spray time of 120 s; Video S4: Wetting behavior of EGaIn liquid metal on CuNPs/PMMA substrate with spray time of 30 s.
