# Supplementary material for: Facile Formation of Metallic Surface with Microroughness via Spray-Coating of Copper Nanoparticles for Enhanced Liquid Metal Wetting
Source: Materials (Basel). 2024 Oct 31;17(21):5299. doi: 10.3390/ma17215299 (PMC11548000; doi:10.3390/ma17215299)
Supplement: Supplementary file 1 [file materials-17-05299-s001.zip › Spray coating_SI-done.pdf]

Supplementary Information

Facile formation of microroughness metallic surface via spray-coating of copper nanoparticles for enhanced liquid metal wetting

Ji-Hye Kim [1], Ju-Hee So [2*] and Hyung-Jun Koo [3*]

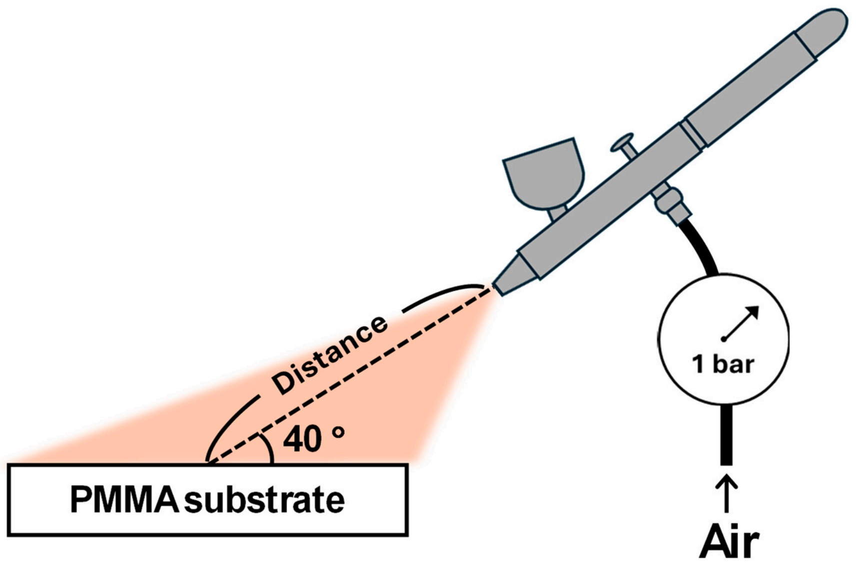

Figure S1. Schematic of the setup for CuNPs spray-coating.

Table S1. Vapor pressure, surface tension, and relative energy difference (RED) of organic solvent used for dispersion.

Solvent	Vapor pressure (mmHg)	Surface tension (mN/m @20 ℃)	RED*
Dichloromethane	355.53 (20 ℃)	26.50	0.58
Chlorobenzene	11 (25 ℃)	33.60	1.00
IPA	44 (25 ℃)	23.00	1.27
DMSO	0.6 (25 ℃)	43.53	0.76
DMF	2.7 (20℃)	37.10	0.73

*RED (Relative Energy Difference) = $Ra/R_{0,PMMA}$, RED < 1 means good solvents and RED >1 means bad solvents.

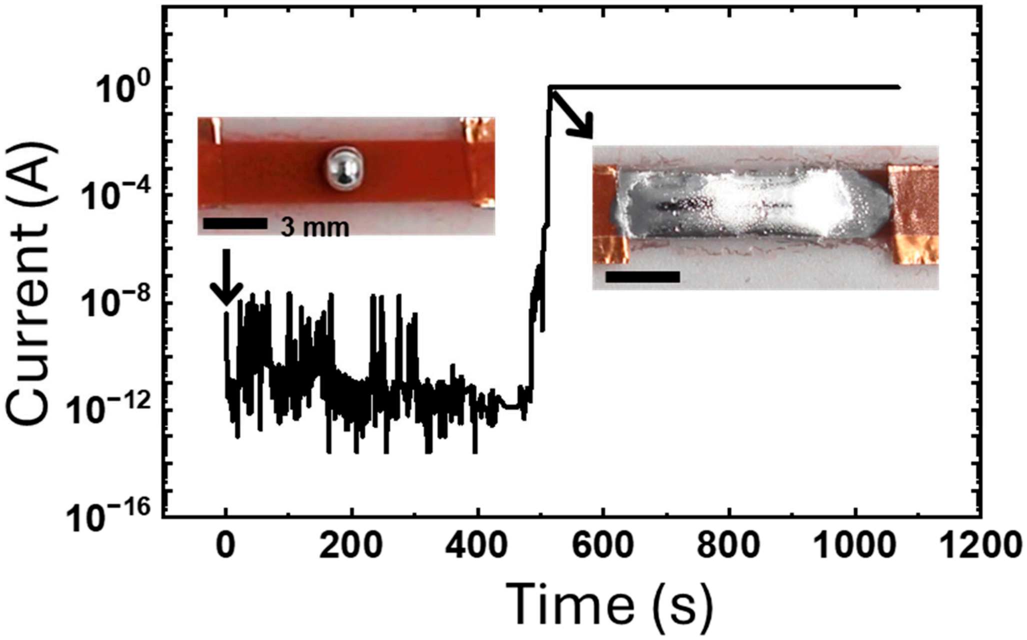

Figure S2. Log plot of change in the current of the spray-coated CuNP film before and after EGaIn wetting.
